# Incidence of systemic inflammatory response syndrome and patient outcome following transcatheter edge-to-edge mitral valve repair

**DOI:** 10.1007/s00392-023-02316-y

**Published:** 2023-10-23

**Authors:** Finn Syryca, Costanza Pellegrini, Marie Gollreiter, Philipp Nicol, N. Patrick Mayr, Hector A. Alvarez-Covarrubias, Niklas Altaner, Tobias Rheude, Stefan Holdenrieder, Heribert Schunkert, Adnan Kastrati, Michael Joner, Erion Xhepa, Teresa Trenkwalder

**Affiliations:** 1 Department of Cardiology, German Heart Center Munich, Technical University of Munich, Munich, Germany; 2 Institute of Anaesthesiology, German Heart Center Munich, Technical University of Munich, Munich, Germany; 3 Institute of Laboratory Medicine, German Heart Center Munich, Technical University of Munich, Munich, Germany; 4 DZHK (German Center for Cardiovascular Research), Partner Site Munich Heart Alliance, Munich, Germany

**Keywords:** SIRS, Transcatheter edge-to-edge mitral valve repair, Inflammation, Mitral valve regurgitation

## Abstract

**Objectives:**

Systemic inflammatory response syndrome (SIRS) is a common finding after cardiovascular interventions. Data on the incidence of SIRS and its impact on outcome in patients undergoing transcatheter edge-to-edge mitral valve repair (MV-TEER) for mitral regurgitation (MR) is lacking.

**Methods:**

From January 2013 to December 2020, 373 patients with moderate or severe MR undergoing MV-TEER were included. SIRS was defined as at least two of the following criteria within 48 h after the procedure: leucocyte count > 12.0 or < 4.0 × 10^9^/l, respiratory rate > 20 breaths per minute or PaCO_2_ ≤ 4.3 kPa/32 mmHg, heart rate > 90 bpm and temperature > 38.0 °C or < 36.0 °C. The primary endpoint was 3-years all-cause mortality.

**Results:**

SIRS was observed in 49.6% (185/373) of patients. Patients who developed SIRS presented more frequently with NYHA III/IV at baseline [SIRS: 82.4% (149/185) vs. no SIRS: 79.0% (147/188); p = 0.029]. Patients who developed SIRS spent more days on ICU (p < 0.001) and overall length of stay was longer (p < 0.001). Relevant residual MR, defined as MR ≥ III in-hospital, was present more often in patients who developed SIRS [SIRS: 11.3% (20/177) vs. no SIRS: 3.93% (7/178), p = 0.036]. At 3 years, all-cause mortality in the entire population was 33.5% (125/373) with an increased all-cause mortality in patients with SIRS compared to patients without SIRS (HR 1.49, [CI 95% 1.04, 2.13]; p = 0.0264). In the multivariate analysis development of SIRS (HR 1.479 [CI 95% 1.016, 2.154]; p = 0.041) was identified as predictor for 3-years all-cause mortality.

**Conclusions:**

SIRS is a common finding after MV-TEER occurring in approximately half of patients. SIRS after MV-TEER was associated with a longer in-hospital stay. In addition, we observed an increased 3-years all-cause mortality in patients with SIRS.

**Graphical abstract:**

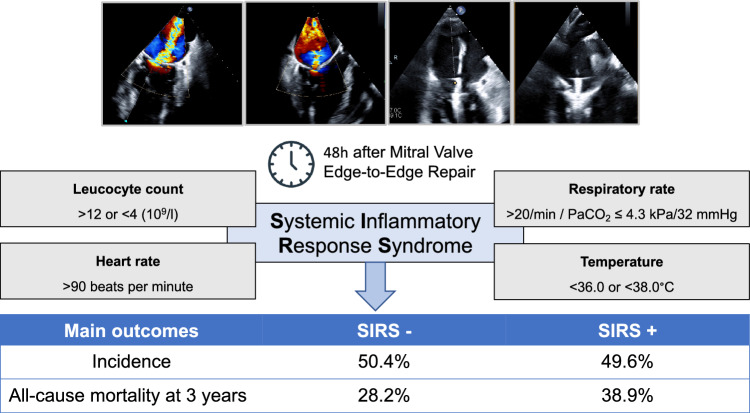

## Introduction

In recent years, transcatheter edge-to-edge mitral valve repair (MV-TEER) has become an established therapeutic strategy for patients with severe, symptomatic mitral valve regurgitation (MR) who are at high or prohibitive risk for surgery [1]. In the context of demographic challenges with a growing number of elderly patients, the amount of MV-TEER procedures is expected to rise even further in the upcoming years [2]. Therefore, the identification of potential periprocedural risk factors and their adequate treatment is crucial to warrant optimal patient management.

The activation of a systemic inflammatory response has been reported in patients after myocardial infarction or coronary artery bypass grafting (CABG) [3–5]. Especially cardiac surgery induces a systemic inflammatory reaction, associated with increased postoperative mortality and morbidity [6, 7]. Squiccimarro et al. observed an incidence of systemic inflammatory response syndrome (SIRS) of 28.3% within 24 h in patients undergoing cardiovascular surgery (including CABG, aortic-, mitral-, and tricuspid valve surgery, surgery of the thoracic aorta, correction of atrial septal defect and ventricular defect or resection of cardiac tumor). In this cohort, SIRS was associated with a more complicated postoperative course and higher postoperative morbidity [7]. In addition, recent studies found that the incidence of SIRS after transcatheter aortic valve implantation (TAVI) was even higher, reaching approximately 40% [8, 9]. Thereby, Sinning et al. showed an increased 1-year mortality in patients who developed SIRS after TAVI, even after excluding patients with periprocedural complications such as major vascular or bleeding complications, and kidney injury [8]. Similar results were observed by Schwietz et al. showing that SIRS was associated with an increased 1-year mortality, but not 30-day mortality [9]. These observations suggest that the adverse outcome of patients with SIRS after TAVI may not be necessarily derived by hospital-associated infections or procedure-related complications.

However, in daily clinical practice, the treating physician is often confronted with the occurrence of postprocedural SIRS without knowing the relevance for the patient and his clinical outcome. Especially, in multimorbid patients undergoing MV-TEER standardized data on the incidence of SIRS and its impact on mortality do not exist so far. Therefore, the present study sought to evaluate the incidence of SIRS and potential prognostic implications of its occurrence in patients undergoing MV-TEER due to severe, symptomatic MR.

## Patients and methods

### Patient population and procedures

All consecutive patients undergoing MV-TEER between January 2013 and December 2020 at the Deutsches Herzzentrum München, Germany, were evaluated for the present retrospective analysis.

In all cases, the indication for MV-TEER was symptomatic moderate-to-severe or severe MR and all procedures were approved by the local heart team. Patient informed consent was obtained prior to each procedure. MV-TEER was performed under general anesthesia and transoesophageal echocardiographic guidance through a transvenous, transseptal approach as previously described [10]. The number of devices implanted was at the discretion of the treating physician. All patients received prophylactic periprocedural antibiotic therapy with a second generation cephalosporin. After the procedure, patients were monitored at the intensive care unit (ICU) for at least 12 h and then transferred to the cardiology ward, remaining under observation for at least 72 h. Clinical, procedural and follow-up data were collected according to a standardized protocol and entered in a central electronic database.

The study was conducted in conformity with the Declaration of Helsinki and the collection of clinical, procedural and follow-up patient data was approved by the local ethics committee.

### Echocardiographic analysis

All echocardiographic studies were performed by experienced institutional cardiologists during clinical routine. Echocardiographic measures were assessed according to the current guideline recommendations [11], and classification of MR severity was performed using a four-group classification (mild≙I°, moderate≙II°, severe≙III°, and massive≙IV°). All patients underwent transthoracic as well as transoesophageal echocardiography before MV-TEER and transthoracic echocardiography after MV-TEER in hospital. Relevant residual MR post procedure was defined as MR ≥ III°.

### Endpoint definition and clinical follow-up

SIRS was defined according to the joint definition of the American College of Chest Physicians/Society of Critical Care Medicine (ACCP/SCCM) Consensus Conference, through the fulfillment of at least two of the following four criteria: leucocyte count > 12.0 or < 4.0 × 10^9^/l, respiratory rate > 20 breaths per minute or PaCO_2_ ≤ 4.3 kPa/32 mmHg, heart rate > 90 beats per minute and temperature > 38.0 °C or < 36.0 °C within 48 h after the procedure [12, 13]. Clinical follow-up including transthoracic echocardiography was routinely performed 30 days and 1 year after MV-TEER. Procedural success was measured after 30 days and was defined according to the definition of the Mitral Valve Academic Research Consortium (MVARC) including the absence of a relevant residual MR [14]. As an elderly patient population was studied, post-procedural 3-years all-cause mortality was defined as a clinically meaningful primary outcome measure. Survival data were obtained from the German Civil Registry, meaning that no patient was lost to follow-up.

### Laboratory methods

Blood samples were obtained pre-procedurally, the same day after the procedure, after 48 h, after 96 h and at discharge, as part of clinical routine. Leucocyte count was measured using fluorescence flow cytometry (Sysmex XN-2000, Kobe, Japan), C-reactive protein (CRP) levels were determined using immunturbidimetry (Cobas c501 Roche, Mannheim, Germany) and lactate dehydrogenase (LDH) levels were assessed using photometry (Cobas c501 Roche, Mannheim, Germany).

### Statistical analysis

Continuous variables are reported as mean ± standard deviation (SD) or median [interquartile range], according to the distribution pattern of the variable, and compared using the Student’s t test or the Mann–Whitney U test as appropriate. Categorical variables are described as frequencies or proportions and compared using the Pearson χ^2^ test (or Fischer’s exact test where any expected cell count of the contingency table was < 5). Event-free survival was estimated by the Kaplan–Meier method and hazard ratios (HR) with two-sided 95% confidence intervals (95% CI) were calculated using the Cox proportional hazards model. A logistic regression computing the odds ratios (OR) with 95% CIs was performed to identify predictors of SIRS. Independent mortality predictors were analyzed by means of multivariable Cox proportional hazards models.

The selection of variables to be included in the multivariable models for mortality at 3 years as well as for SIRS occurrence was performed using the LASSO (Least Absolute Shrinkage and Selection Operator) regression method [15], after entering all relevant clinical, echocardiographic and laboratory parameters as well as relevant medications as candidates.

A 2-sided p value of < 0.05 was considered statistically significant for all analyses. Statistical analysis was performed using the R 4.10 Statistical Package (R Foundation for Statistical Computing, Vienna, Austria).

## Results

### Patient population

**Fig. 1:**
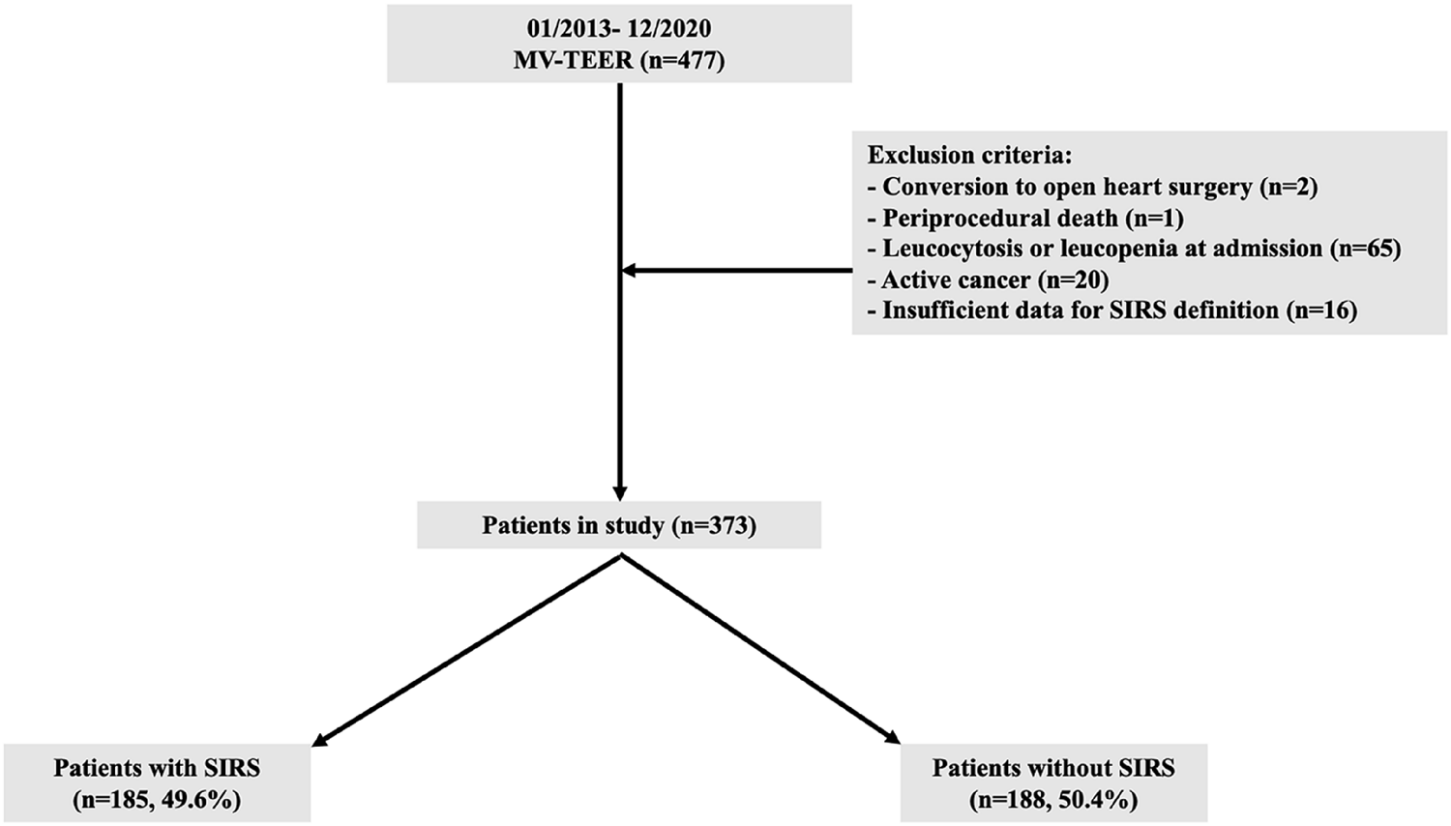
Study population and detailed exclusion criteria applied. *MV-TEER* transcatheter edge-to-edge mitral valve repair, *SIRS* systemic inflammatory response syndrome

A total of 477 consecutive patients underwent MV-TEER at the Deutsches Herzzentrum München, Germany, between January 2013 and December 2020. Of these, patients with conversion to open heart surgery (n = 2), periprocedural death (n = 1), leucocytosis or leucopenia at admission (n = 65), active cancer (n = 20) and insufficient data to apply SIRS definition (n = 16) were excluded, resulting in a final study population of 373 patients. A detailed flow chart with exclusion criteria is depicted in Fig. 1.

Table 1 shows baseline characteristics of the patient population according to the presence/absence of SIRS. Overall, mean age was 79.0 years [interquartile range [IQR] 73.0, 82.0], 42.9% (160/373) were female and median logistic EuroSCORE I was 13.9% [IQR 8.9, 22.2]. Patients who developed SIRS presented more frequently with NYHA class III/IV (SIRS: 82.4% (149/185), no SIRS: 79% (147/188); p = 0.029) and less frequently with previous coronary artery disease (CAD) (SIRS: 60.0% (111/185), no SIRS: 70.7% (133/188); p = 0.038). Interventricular septum diameter (IVSD) was smaller in patients who developed SIRS (SIRS: 11.0 mm [9.53, 12.0], no SIRS: 11.0 mm [10.0, 13.0]; p = 0.025) with a trend towards a reduced left and right ventricular function.

**Table 1:** Baseline characteristics

	All patients n = 373	No SIRS n = 188	SIRS n = 185	p value
Age, years	79.0 [73.0, 82.0]	79.0 [73.0, 82.0]	78.0 [72.0, 82.0]	0.717
Female gender	160 (42.9)	72 (38.3)	88 (47.6)	0.088
EuroSCORE I, %	13.9 [8.9, 22.2]	13.8 [8.72, 22.9]	14.1 [8.96, 22.0]	0.833
EuroSCORE II, %	5.53 [3.47, 9.72]	5.38 [3.20, 8.68]	5.66 [3.81; 10.9]	0.170
NYHA III/IV	296 (80.6%)	147 (79%)	149 (82.4)	**0.029**
Arterial hypertension	318 (85.3)	167 (88.8)	151 (81.6)	0.145
Hypercholesterolemia	223 (59.8)	120 (63.8)	103 (55.7)	0.134
Diabetes mellitus	77 (20.6)	45 (23.9)	32 (17.3)	0.145
COPD	52 (13.9)	24 (12.8)	28 (15.1)	0.609
Previous CAD	244 (65.4)	133 (70.7)	111 (60.0)	**0.038**
Previous CABG	52 (13.9)	32 (17.0)	20 (10.8)	0.114
Previous valve surgery	55 (14.7)	28 (14.9)	27 (14.6)	1.000
Previous stroke/TIA	50 (13.41)	29 (15.39)	21 (11.35)	0.518
Previous dialysis	10 (2.68)	2 (1.06)	8 (4.32)	0.056
History of atrial fibrillation	113 (30.3)	57 (30.3)	56 (30.3)	1.000
Carotid stenosis	28 (7.51)	11 (5.85)	17 (9.19)	0.305
Laboratory findings				
eGFR, ml/min/1.73 m^2^	47.0 [35.0, 59.2]	49.0 [37.0, 60.0]	46.0 [34.0, 59.0]	0.274
NT-proBNP, ng/l	3360 [1595, 7685]	2900 [1400, 5980]	3810 [1865, 8115]	0.167
Hemoglobin, g/dl	12.6 [11.2, 13.5]	12.6 [11.2, 13.6]	12.5 [11.2, 13.4]	0.550
Heart failure medication				
ARNI	34 (9.29)	18 (9.73)	16 (8.84)	0.910
ACE-inhibitor/RAAS-blocker	281 (75.7)	141 (75.4)	140 (76.1)	0.974
Beta-blocker	310 (83.6)	156 (83.4)	154 (83.7)	1.000
Diuretics/MRA	348 (93.8)	174 (93.0)	174 (94.6)	0.696
Echocardiographic findings				
Left ventricular EF, %	45 [30.0, 60.0]	48.0 [32.0, 60.0]	44.0 [30.0, 58.0]	0.093
Mitral valve pathology				0.632
Functional regurgitation^a^	217 (58.2)	108 (57.5)	109 (58.9)	
Structural regurgitation^b^	109 (29.2)	57 (30.3)	52 (28.1)	
Complex^c^	47 (12.6)	23 (12.2)	24 (13.0)	
Mitral valve mean gradient, mmHg	2.00 [1.00, 2.00]	2.00 [1.00, 2.00]	2.00 [1.00, 2.00]	0.304
LVEDD, mm	57.0 [51.0, 63.0]	57.0 [52.0, 64.0]	57.0 [49.0, 62.0]	0.361
LVESD, mm	43.0 [34.0, 52.0]	42.5 [34.0, 53.2]	44.0 [34.0, 51.0]	0.946
IVSD, mm	11.0 [10.0, 13.0]	11.0 [10.0, 13.0]	11.0 [9.53, 12.0]	**0.025**
PAP, mmHg	48.3 [40.0, 62.0]	48.3 [40.0, 63.0]	48.5 [40.0, 59.8]	0.543
TAPSE, mm	17.0 [13.0, 20.0]	17.0 [14.0, 21.0]	16.5 [13.0, 20.0]	0.058

Bold indicates statistically significant *p* values

Data are median [interquartile range] or n (%)

*ACE-Inhibitor* angiotensin-converting enzyme-inhibitor, *AML* anterior mitral leaflet, *CAD* coronary artery disease, *ARNI* angiotensin receptor-neprilysin-inhibitor, *CABG* coronary artery bypass grafting, *COPD* chronic obstructive pulmonary disease, *EF* ejection fraction, *eGFR* estimated glomerular filtration rate, *IVSD* interventricular septum diameter, *LVEDD* left ventricular enddiastolic diameter, *LVESD* left ventricular endsystolic diameter, *MRA* mineralcorticoid receptor antagonist, *NT-proBNP* N-Terminal pro-hormone Brain Natriuretic Peptide, *NYHA* New York Heart Association Classification, *PAP* pulmonary artery pressure, *PML* posterior mitral leaflet, *RAAS-blocker* renin angiotensin aldosterone system-blocker, *SIRS* systemic inflammatory response syndrome, *TAPSE* tricuspid annular plane systolic excursion, *TIA* transient ischemic attack

^a^Ventricular dilatation, atrial dilatation or atrial/ventricular dilatation

^b^AML prolaps, PML prolaps or prolaps both/Morbus Barlow

^c^Complex phenotype including patients post endocarditis, second clip procedure, mitral valve reconstruction and others

### Incidence of systemic inflammation response syndrome after transcatheter edge-to-edge mitral valve repair

**Fig. 2:**
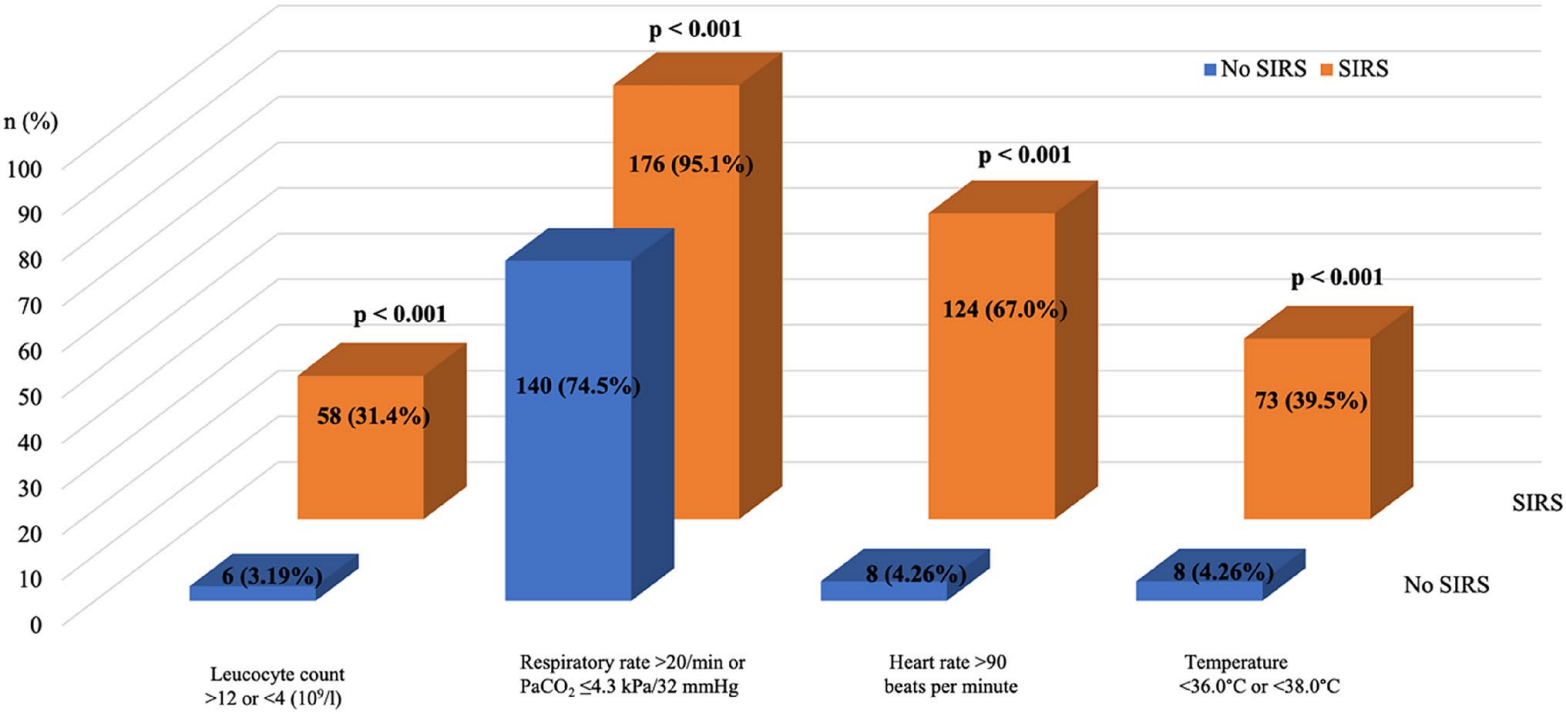
Drivers of SIRS definition in patients after MV-TEER. °*C* Celsius, *min* minute, *MV-TEER* transcatheter edge-to-edge mitral valve repair, *SIRS* systemic inflammatory response syndrome

Overall, the incidence of SIRS after transcatheter edge-to-edge mitral valve repair was 49.6% (185/373). The four different drivers of SIRS definition are shown in Fig. 2.

### Inflammatory biomarkers following MV-TEER

**Fig. 3:**
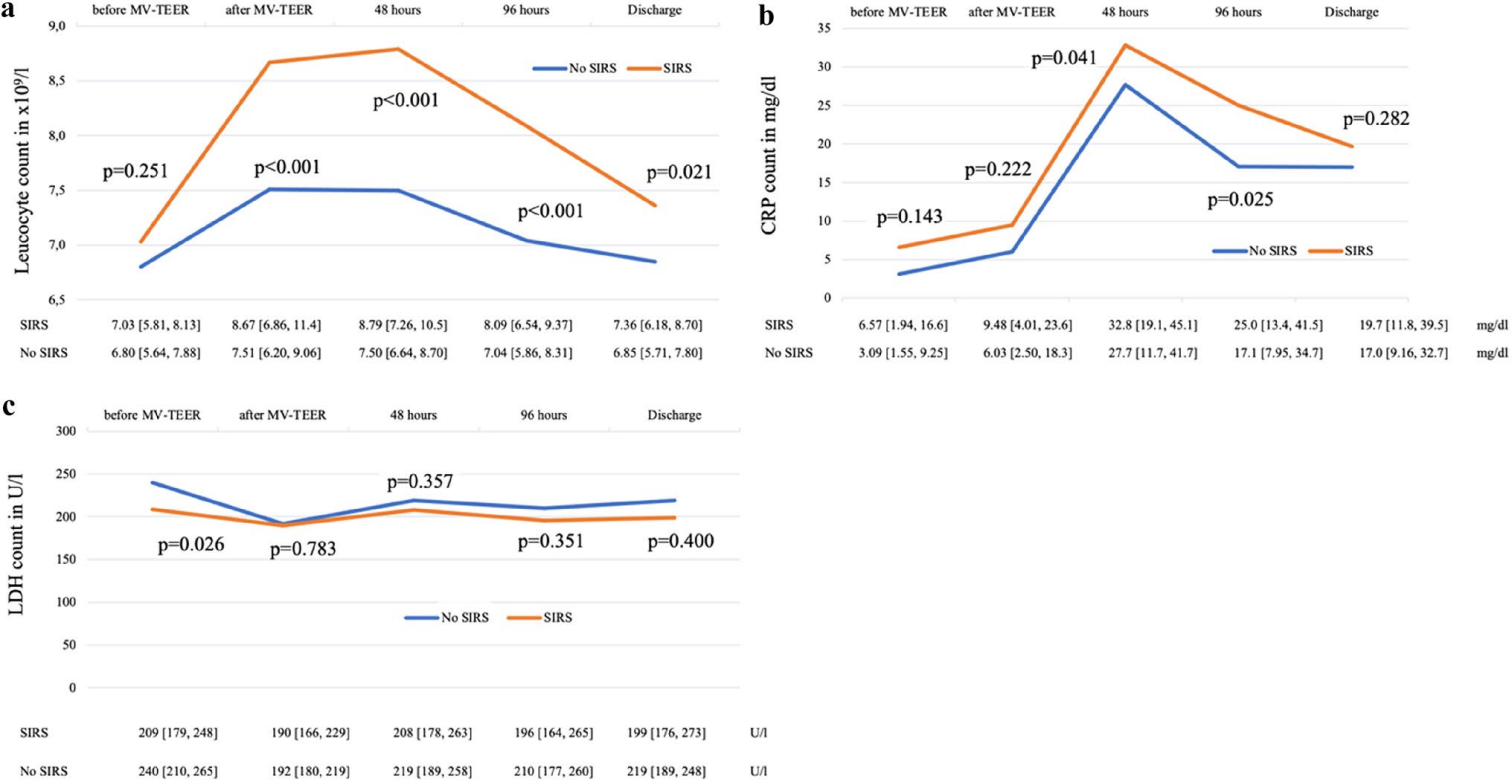
Development of inflammatory markers before and after MV-TEER; leucocytes (**a**), CRP (**b**) and LDH (**c**). *CRP* C-reactive protein, *LDH* lactate dehydrogenase, *MV-TEER* transcatheter edge-to-edge mitral valve repair, *SIRS* systemic inflammatory response syndrome

The time course of inflammatory biomarkers of interest at admission as well as following MV-TEER are depicted in Fig. 3. Inflammatory biomarkers increased after the procedure, irrespective of SIRS development: leucocyte count increased in both groups after MV-TEER, however this effect was more pronounced in patients which experienced SIRS (leucocyte count at 48 h in patients with SIRS: 8.79 × 10^9^/l [7.26, 10.5] vs. no SIRS: 7.50 × 10^9^/l [6.64, 8.70]; p < 0.001) (Fig. 3a). A similar effect was observed regarding the increase in CRP levels (CRP count at 48 h in patients with SIRS: 32.8 mg/dl [19.1, 45.1], no SIRS: 27.7 mg/dl [11.7, 41.7]; p = 0.041) (Fig. 3b). In contrast, LDH levels did not differ significantly (LDH count at 48 h in SIRS: 208 U/l [178, 263], no SIRS: 219 U/l [189, 258]; p = 0.357) (Fig. 3c).

### Impact of SIRS on clinical outcome

Procedural characteristics and in-hospital complications are shown in Table 2. Relevant residual MR after MV-TEER was present more often in patients developing SIRS versus patients without SIRS (SIRS 11.30% (20/177) vs. no SIRS: 3.93% (7/178), p = 0.036). Patients with SIRS after MV-TEER spent more days on ICU (SIRS: 1.00 [1.00, 2.00] days vs. no SIRS: 1.00 [1.00, 1.00] days; p < 0.001) and had an overall longer length of stay than patients who did not develop SIRS (SIRS: 3.00 [3.00, 5.00] days vs. no SIRS group: 3.00 [3.00, 4.00] days; p < 0.001). Differences in length of stay and days on ICU remained significant despite dividing the number of days into smaller subgroups (Table 2). Interestingly, relevant residual MR, defined as MR ≥ III in-hospital, was present more often in patients who developed SIRS (SIRS 11.30% (20/177) vs. no SIRS: 3.93% (7/1178), p = 0.036). In addition, patients with SIRS had required red blood cell (RBC) transfusions more frequently (SIRS: 9.44% (17/180) vs. no SIRS: 2.69% (5/186; p = 0.012).

**Table 2:** Procedural characteristics and in-hospital outcome

	All patients n = 373	No SIRS n = 188	SIRS n = 185	p value
Procedural characteristics				
Procedural time, min	95.0 [74.0, 126.0]	92.5 [74.0, 118.0]	97.0 [74.0, 135]	0.171
Fluoro time, min	13.4 [9.6, 21.4]	12.9 [9.25, 19.6]	14.4 [9.85, 23.5]	0.110
In-hospital outcome				
Lifethreatening bleeding	4 (1.07)	0 (0.00)	4 (2.16)	0.060
Major vascular complication	6 (1.61)	2 (1.06)	4 (2.16)	0.455
Relevant residual MR	27 (7.61)	7 (3.93)	20 (11.3)	**0.036**
Days on ICU	1.00 [1.00, 1.00]	1.00 [1.00, 1.00]	1.00 [1.00, 2.00]	** < 0.001**
Days ≤ 1	294 (78.8)	164 (87.2)	130 (70.3)	** < 0.001**
Days 1–3	58 (15.5)	21 (11.2)	37 (20.0)	** < 0.001**
Days > 3	21 (5.63)	3 (1.60)	18 (9.73)	** < 0.001**
Length of stay, days	3.00 [3.00, 4.00]	3.00 [3.00, 4.00]	3.00 [3.00, 5.00]	** < 0.001**
Days ≤ 3	202 (54.2)	117 (62.2)	85 (45.9)	** < 0.001**
Days 3–7	142 (38.1)	66 (35.1)	76 (41.1)	** < 0.001**
Days > 7	29 (7.77)	5 (2.66)	24 (13.0)	** < 0.001**
Red blood cell transfusion	22 (6.01)	5 (2.69)	17 (9.44)	**0.012**

Bold indicates statistically significant *p* values

Data are mean ± standard deviation, median [interquartile range] or n (%). Data on Red Blood Cell Transfusion was available for 366 patients (SIRS n = 180, no SIRS = 186; p = 0.281)

*MR* mitral regurgitation, *ICU* intensive care unit, *RIFLE* risk-injury-failure-loss-end stage renal disease, *SIRS* systemic inflammatory response syndrome

Regarding procedural success at 30 days there was no significant difference between patients with SIRS (70.8%; 119/168) and patients without SIRS (79.2%; 137/173), p = 0.097). Functional recovery of patients after MV-TEER showed numerically more patients with NYHA Class ≥ III at 30 days in the SIRS group (30.4%; 45/148) versus 21.6% (33/153) in the no SIRS group, yet without reaching statistical significance (p = 0.255), (Table 3). Further, NYHA Class ≥ III at 1 year was 35.0% in the SIRS group (36/103) and 28.4% in the no SIRS group (35/123); (p = 0.459). Interestingly, in the multivariate analysis a higher left ventricular ejection fraction at baseline showed a lower risk of SIRS occurrence (OR 0.977 [95% CI: 0.958, 0.977]; p = 0.030), (Table 4).

**Table 3:** Follow-up

	All patients n = 373	No SIRS n = 188	SIRS n = 185	p value
Procedural success^a^	256 (75.1)	137 (79.2)	119 (70.8)	0.097
New arrhythmia at 30 days	32 (8.65)	16 (8.56)	16 (8.74)	1.000
NYHA ≥ III at 30 days	78 (25.93)	33 (21.61)	45 (30.43)	0.255
NYHA ≥ III at 1 year	71 (31.44)	35 (28.41)	36 (35.00)	0.459

Bold indicates statistically significant *p* values

Data are median [interquartile range] or n (%). Procedural success was available for 341 patients (SIRS n = 168 vs. no SIRS n = 173, p = 0.816). New arrhythmia was available for 370 patients (SIRS n = 183 vs. no SIRS n = 187; p = 0.620). NYHA Class was available for 301 patients at 30 days (SIRS n = 148 vs. no SIRS = 153; p = 0.836) and 226 patients after 1 year (SIRS n = 103 vs. no SIRS = 123; p = 0.069)

*NYHA* New York Heart Association Classification, *SIRS* systemic inflammatory response syndrome

^a^Defined according to MVARC criteria [14]

**Table 4:** Multivariate analysis for the development of SIRS

	Odds ratio [95% Confidence interval]	p value
Age, years	0.997 [0.961, 1.033]	0.872
Female	1.467 [0.912, 2.370]	0.114
Arterial hypertension	0.676 [0.353, 1.274]	0.230
Hypercholesterolemia	0.889 [0.551, 1.436]	0.630
Diabetes	0.697 [0.402, 1.200]	0.195
COPD	1.163 [0.624, 2.180]	0.633
Previous CAD	0.707 [0.431, 1.155]	0.167
Previous CABG	0.685 [0.345, 1.334]	0.271
Previous valve surgery	1.100 [0.580, 2.091]	0.768
Previous stroke/TIA	0.814 [0.565, 1.162]	0.262
History of atrial fibrillation	1.016 [0.624, 1.654]	0.948
eGFR, ml/min/1.73 m^2^	1.000 [0.987, 1.014]	0.920
Hemoglobin, g/dl	0.945 [0.821, 1.085]	0.424
Left ventricular EF, %	0.977 [0.958, 0.997]	**0.030**
Diuretics/MRA	1.591 [0.652, 3.99]	0.309
Functional mitral valve regurgitation	0.829 [0.445, 1.537]	0.553
Complex mitral valve regurgitation	1.002 [0.470, 2.131]	0.995
PAP, mmHg	0.997 [0.985, 1.010]	0.729

Bold indicates statistically significant *p* values

*CAD* coronary artery disease, *CABG* coronary artery bypass grafting, *COPD* chronic obstructive pulmonary disease, *NYHA* New York Heart Association Classification, *EF* ejection fraction, *eGFR* estimated glomerular filtration rate, *MRA* mineralcorticoid receptor antagonist, *PAP* pulmonary artery pressure, *SIRS* systemic inflammatory response syndrome, *TIA* transient ischemic attack

**Fig. 4:**
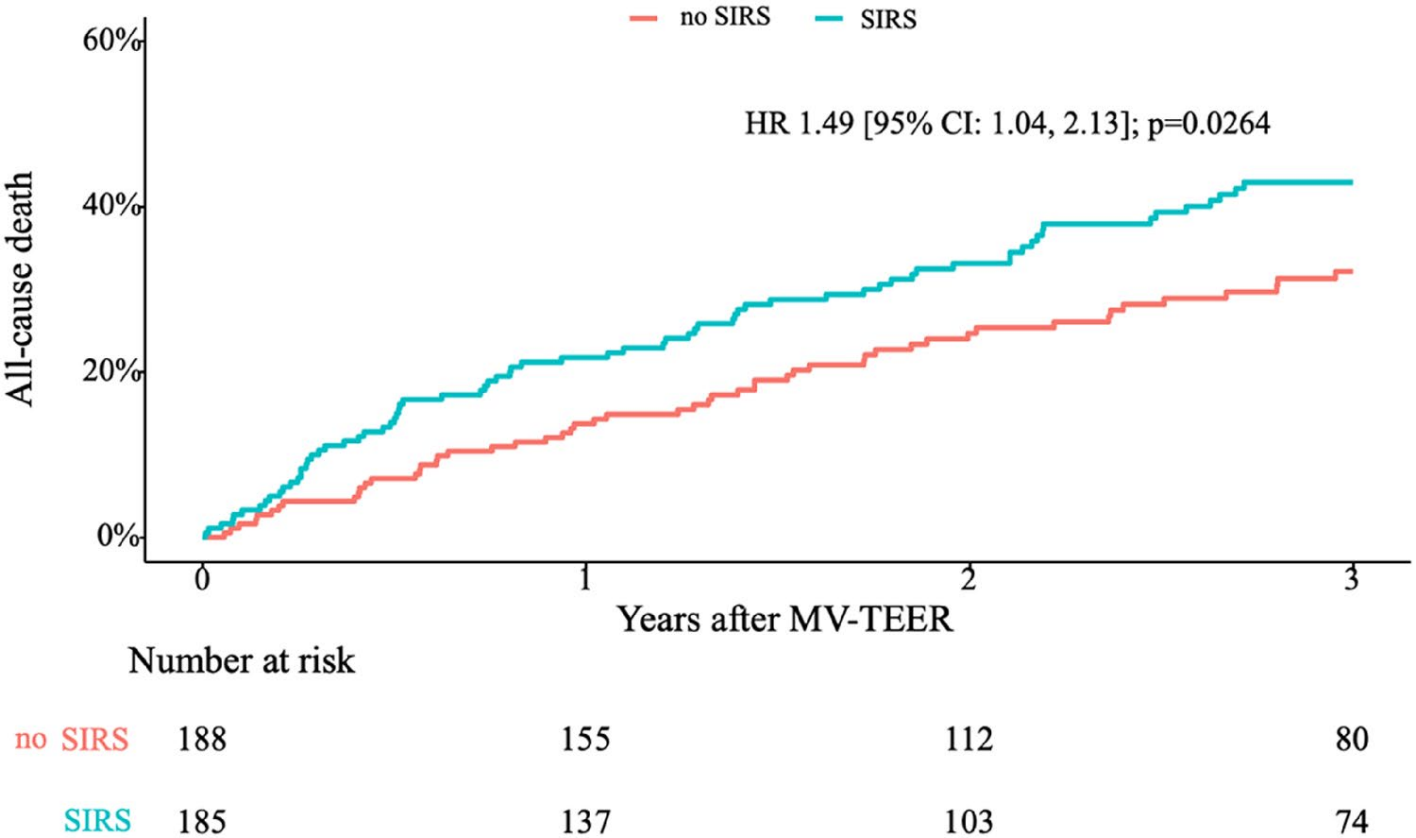
Influence of SIRS on all-cause mortality at 3 years in patients after MV-TEER. *MV-TEER* transcatheter edge-to-edge mitral valve repair, *SIRS* systemic inflammatory response syndrome

Overall, all-cause mortality at 3 years was 33.5% (125/373) in the study population. We observed a significantly higher 3-year mortality in patients who developed SIRS after MV-TEER (HR 1.49, [95% CI: 1.04, 2.13]; p = 0.0264) compared to patients who did not develop SIRS (SIRS: 38.9% (72/185) vs. SIRS: 28.2% (53/188); p = 0.03) (Fig. 4).

When analyzed in a multivariable cox regression analysis, the development of SIRS (HR 1.479 [95% CI 1.016, 2.154]; p = 0.041), previous stroke/TIA (HR 1.342 [95% CI 1.023, 1.760]; p = 0.033) and use of diuretics (HR 3.990 [95% CI 1.228, 12.965]; p = 0.021) were identified as independent risk factors for 3-years all-cause mortality (Table 5). Increased eGFR (HR 0.973 [95% CI 0.959, 0.987]; p = 0.0001), hemoglobin (HR 0.758 [95% CI 0.667, 0.860]; p < 0.0001), ejection fraction (HR 0.979 [95% CI 0.963, 0.996]; p = 0.016) and concomitant CAD (HR 0.591 [95% CI 0.394, 0.887]; p = 0.011) showed a lower risk for 3-years all-cause mortality.

**Table 5:** Multivariate analysis for all-cause mortality at 3-years

	Hazard ratio [95% confidence interval]	p value
SIRS	1.479 [1.016, 2.154]	**0.041**
Age, years	1.016 [0.985, 1.049]	0.303
Female	0.677 [0.447, 1.025]	0.065
Arterial hypertension	0.783 [0.454, 1.350]	0.380
Hypercholesterolemia	0.845 [0.573, 1.247]	0.398
Diabetes	1.017 [0.654, 1.582]	0.937
COPD	1.065 [0.632, 1.793]	0.811
Previous CAD	0.591 [0.394, 0.887]	**0.011**
Previous CABG	1.482 [0.828, 2.650]	0.184
Previous valve surgery	1.484 [0.895, 2.460]	0.125
Previous stroke/TIA	1.342 [1.023, 1.760]	**0.033**
History of atrial fibrillation	0.993 [0.645, 1.529]	0.977
eGFR, ml/min/1.73 m^2^	0.973 [0.959, 0.987]	**0.0001**
Hemoglobin, g/dl	0.758 [0.667, 0.860]	** < 0.0001**
Left ventricular EF, %	0.979 [0.963, 0.996]	**0.016**
Diuretics/MRA	3.990 [1.228, 12.965]	**0.021**
Functional mitral valve regurgitation	0.852 [0.488, 1.488]	0.574
Complex mitral valve regurgitation	1.437 [0.776, 2.660]	0.248
PAP, mmHg	0.989 [0.979, 1.001]	0.077

Bold indicates statistically significant *p* values

*CAD* coronary artery disease, *CABG* coronary artery bypass grafting, *COPD* chronic obstructive pulmonary disease, *NYHA* New York Heart Association Classification, *EF* ejection fraction, *eGFR* estimated glomerular filtration rate, *MRA* mineralcorticoid receptor antagonist, *PAP* pulmonary artery pressure, *SIRS* systemic inflammatory response syndrome, *TIA* transient ischemic attack

## Discussion

This retrospective study is the first systematic analysis of the incidence of SIRS in patients undergoing MV-TEER. We found that the incidence of SIRS within 48 h after MV-TEER was high, affecting approximately half of the patients. Inflammatory markers increased in all groups irrespective of SIRS, but were significantly higher in patients with SIRS. The development of SIRS after MV-TEER occurred more often in patients with relevant residual MR and was associated with a prolonged in-hospital stay. Furthermore, we observed an increased all-cause mortality at 3 years in patients with postprocedural SIRS.

## Potential mechanisms of systemic inflammatory response syndrome

SIRS is a common clinical finding in patients undergoing cardiac surgery [16, 17]. Procedure-related organ hypoperfusion leading to regional ischemia followed by reperfusion is a known trigger for the release of immune mediators and has been described as contributor in SIRS development after CABG [18]. Furthermore, the surgical trauma during CABG might also stimulate the immune response [3, 18, 19]. Similar, SIRS is known to occur in patients after myocardial infarction and comparably to the ischemia–reperfusion-triggered cytokine release in CABG, concentrations of inflammatory cytokines correlate with the blood flow through the infarct-related artery [20]. Additionally, SIRS has also been observed in patients after TAVI [8, 9, 21]. Possible pathomechanisms were reported to be suboptimal organ perfusion caused by transient drop in total or regional blood flow with consecutive hypotension during rapid pacing, valve deployment, post-dilatation as well as vascular complications and/or major bleeding events [8]. In our study there was no difference between patients with and without SIRS regarding the procedural time, which can be used as indicator for procedure-related ischemia. Also, periprocedural complications including vascular complications and major bleedings were similar between patients with and without SIRS. However, RBC transfusion rates were higher in patients who developed SIRS. RBC transfusion can cause the co-administration of e.g. interleukin (IL)-8 which accumulates in stored RBC packages and can contribute to the development of pyrexia and leucocytosis [8, 22].

Furthermore, emerging hypotheses suggest a shear-stress induced development of SIRS in patients after TAVI. Underlying considerations are based on the anatomy of the aortic valve leaflet, composed of two different cell types, an interstitial layer of fibroblast-like cells named valve interstitial cells (VICs), and two outer-layers of valve endothelial cells (VECs) [23]. By tissue damage, e.g. through TAVI procedure-related dilatation of the aortic annulus, VICs become activated to myofibroblasts, thus leading to the production and secretion of a number of cytokines within tissue repair [24, 25], contributing to the development of SIRS. However, the impact of periprocedural-induced shear stress with regard to clinical outcomes remains a matter of debate and needs to be investigated in further studies.

## Systemic inflammatory response syndrome in transcatheter edge-to-edge mitral valve repair

So far, SIRS has not been investigated in patients undergoing MV-TEER. Applying the current knowledge of SIRS development to an edge-to edge repair of the mitral valve, a potential procedure-related trigger for SIRS could be the grasping of the mitral valve leaflets. This step during MV-TEER followed by the closing of the transcatheter device causes a significant mechanical stress on the mitral valve leaflets and its apparatus and could be a pendant to the aortic stretch during TAVI.

Another important aspect to consider is that MV-TEER cohorts typically comprise mainly old and multimorbid patients. Nowadays, the elderly population (> 65 years old) in Europe represents 19.7% of the population and is expected to reach 28.5% in 2050 [26]. Biologically, aging is associated with a physiological process of tissue degeneration related to chronic inflammation [27]. This mechanism of age-related chronic inflammation is called “inflammaging”, which was initially defined as progressive increase of proinflammation in aged organisms [28], leading to increased morbidity and mortality [29–32]. Despite lacking evidence on the direct interaction between “inflammaging” and the development of postprocedural SIRS, “inflammaging” may represent an additional risk factor in elderly, multimorbid patients. In our cohort, we observed an increase of serum inflammatory markers (CRP, leucocytes) in both, the SIRS group and no SIRS group; however, this effect was more pronounced in the SIRS group.

Besides the above-mentioned mechanisms, a further possible contribution to the development of SIRS might be the standard performance of general anesthesia (GA) in an elderly, already frail patient collective. GA is reported to increase stress hormone release by its invasive character, thus accelerating inflammatory processes [21]. However, a recent meta-analysis, including four studies comparing GA with deep sedation (DS) after MV-TEER, showed no difference regarding the composite endpoint of all-cause death, stroke, pneumonia or major to life-threatening bleeding between patients treated with DS as compared to GA, while ICU stay was longer after GA compared to DS [33].

In addition, anaesthesiologic monitoring in patients undergoing MV-TEER is challenging as patients usually are hypovolemic due to a continuous diuretic therapy. Nearly all commonly used anesthetic agents which are used for general anesthesia for MV-TEER procedure lead to vasodilatation resulting in hypotension which has to be treated with a vasopressor. Thereby, increased systemic vascular resistance can increase MV regurgitation, resulting in mandatory inotropic support in patients with a reduced left-ventricular ejection fraction, to counteract the hemodynamic results of a reduced MV-regurgitation after MV-TEER. Interestingly, in our study residual MR was associated with SIRS whereas patients with a higher ejection fraction showed a lower risk for developing SIRS. Thus, the hemodynamic state of the patient during the procedure may be a relevant factor in SIRS development after MV-TEER [34, 35].

## Incidence of systemic inflammatory response syndrome and its impact on patient outcome

The occurrence of SIRS after various cardiac interventions, including CABG and TAVI, has been previously described with high incidences of up to 40% [7–9]. In our study, we found an even higher incidence of SIRS after MV-TEER, in nearly half of the patients (49.6%). This might be second to differences regarding the standard patient collectives assessed for the respective procedures: While TAVI is increasingly performed in younger patients with intermediate or even low surgical risk [36, 37], patients assessed for MV-TEER are usually multimorbid with high or prohibitive surgical risk [1].

Despite the finding of a high SIRS incidence after MV-TEER, we additionally observed that patients who developed SIRS had a prolonged stay on intensive care unit and overall, a longer in-hospital stay. Further, we observed that development of SIRS after MV-TEER increases 3-years all-cause mortality, which was high, at 33.5% and is in line with previous studies such as the COAPT trial, which showed a mortality of 32.7% at 2 years [38]. Interestingly, we found that CAD was associated with a lower risk of 3-years all-cause mortality in our cohort. Although only speculative, one explanation for this finding may be that patients with CAD develop relevant MR due to regional wall motion abnormalities and not due to a global reduced left ventricular ejection fraction as for example seen in advanced stages of dilated cardiomyopathy. In addition, use of diuretics was a strong predictor of mortality pointing to advanced stages of MR and congestive heart failure. In accordance with this, higher eGFR, hemoglobin and increased left ventricular ejection fraction were predictors of a reduced 3-years mortality.

Furthermore, we found SIRS to be a predictor for all-cause mortality after 3 years. However, these results have to be interpretated carefully and further studies are necessary to better characterize patients that develop SIRS and also to provide further insight into the underlying pathomechanism.

## Limitations

All limitations inherent to retrospective data analysis also apply to our study. Especially, causality between the development of SIRS and clinical outcome cannot be established from such analysis and requires prospectively designed randomized trials. In particular, a potential influence of periprocedural catecholamine administration and ventilation time on the development of SIRS cannot be excluded. Furthermore, adding an external cohort would have improved our analysis by increasing the sample size. In addition, we analysed a cohort of multimorbid and old patients and therefore the results cannot be extrapolated to a younger, healthier cohort. However, given the globally growing numbers of MV-TEER procedures and transcatheter valve interventions in general, further investigations addressing causes and pathomechanisms of SIRS are needed in order to derive preventive and therapeutic consequences to optimize periprocedural management of patients at risk.

## Conclusion

SIRS development is a common finding in patients undergoing MV-TEER for MR and is associated with a prolonged in-hospital stay. SIRS development after MV-TEER suggests an adverse impact on patient outcome, however the underlying pathomechanims have to be evaluated further to allow optimized patient management.

## Data Availability

Data available on request.

## Abbreviations

CRP: C-reactive protein
LDH: Lactate dehydrogenase
MR: Mitral valve regurgitation
MV-TEER: Transcatheter edge-to-edge mitral valve repair
SIRS: Systemic inflammatory response syndrome
